# Analysis of the characteristics and expression profiles of coding and noncoding RNAs of human dental pulp stem cells in hypoxic conditions

**DOI:** 10.1186/s13287-019-1192-2

**Published:** 2019-03-12

**Authors:** Ruitang Shi, Haoqing Yang, Xiao Lin, Yangyang Cao, Chen Zhang, Zhipeng Fan, Benxiang Hou

**Affiliations:** 1 Department of Endodontics, Beijing Stomatological Hospital, School of Stomatology, Capital Medical University, Beijing, China; 2 Laboratory of Molecular Signaling and Stem Cells Therapy, Beijing Key Laboratory of Tooth Regeneration and Function Reconstruction, Beijing Stomatological Hospital, School of Stomatology, Capital Medical University, Beijing, China; 3 Department of Implant Dentistry, Beijing Stomatological Hospital, School of Stomatology, Capital Medical University, Beijing, China

**Keywords:** Hypoxia, Dental pulp stem cells (DPSCs), Coding RNA, Long noncoding RNA (lncRNA), MicroRNA (miRNA)

## Abstract

**Background:**

Human dental pulp stem cell (DPSC)-mediated regenerative endodontics is a promising therapy for damaged teeth; however, the hypoxic environment in root canals can affect tissue regeneration. In this study, we investigate the characteristics and possible regulatory mechanisms of DPSC function under hypoxic conditions.

**Methods:**

Human DPSCs were cultured under normoxia (20% O_2_) and hypoxia (3% O_2_). DPSC proliferation and osteo/odontogenic differentiation potential were assessed by Cell Counting Kit-8 (CCK8) assay, carboxyfluorescein succinimidyl ester (CFSE) assay, alkaline phosphatase (ALP) activity, Alizarin red staining, real-time RT-PCR assays, and western blot analysis. Microarray and bioinformatic analyses were performed to investigate the differences in the mRNA, lncRNA, and miRNA expression profiles of DPSCs.

**Results:**

DPSCs exhibited a more powerful proliferation ability and lower osteo/odontogenic differentiation potential in hypoxic conditions. A total of 60 mRNAs (25 upregulated and 35 downregulated), 47 lncRNAs (20 upregulated and 27 downregulated), and 14 miRNAs (7 upregulated and 7 downregulated) in DPSCs were differentially expressed in the hypoxia group compared with the normoxia group. Bioinformatic analysis identified that 7 mRNAs (GRPR, ERO1L, ANPEP, EPHX1, PGD, ANGPT1, and NQO1) and 5 lncRNAs (AF085958, AX750575, uc002czn.2, RP3-413H6.2, and six-twelve leukemia (STL)) may be associated with DPSCs during hypoxia according to CNC network analysis, while 28 mRNAs (including GYS1, PRKACB, and NQO1) and 13 miRNAs (including hsa-miR-3916 and hsa-miR-192-5p) may be involved according to miRNA target gene network analysis. The depletion of one candidate lncRNA, STL, inhibited the osteo/odontogenic differentiation potentials of DPSCs.

**Conclusions:**

Our results revealed that hypoxia could enhance the proliferation ability and impair the osteo/odontogenic differentiation potential of DPSCs in vitro. Furthermore, our results identified candidate coding and noncoding RNAs that could be potential targets for improving DPSC function in regenerative endodontics and lead to a better understanding of the mechanisms of hypoxia’s effects on DPSCs.

**Electronic supplementary material:**

The online version of this article (10.1186/s13287-019-1192-2) contains supplementary material, which is available to authorized users.

## Background

Pulpitis and periapical periodontitis are the most prevalent oral diseases, and root canal treatment is the current primary treatment. Although the treatment has been shown to be highly effective, after treatment, teeth can become devitalized, brittle, and susceptible to fracture, leading to a higher incidence of extraction [1, 2]. During the past decade, advanced discoveries in cell-based therapies have provided novel insights into tooth regeneration. Regenerative endodontics (RE) is a clinical procedure that aims to regenerate the dentin-pulp complex (DPC) inside root canals [3]. The DPC can provide nutrition and biological barriers for teeth, prevent the invasion of bacteria, reduce the risk of tooth fracture, and extend the life of a tooth. RE is a more ideal treatment for teeth that are damaged from pulpitis and periapical periodontitis, which is garnering increased attention [4–6]. Human dental pulp stem cells (DPSCs), a type of mesenchymal stem cell (MSC), are isolated from dental pulp and are ideal seeding cells for RE. A previous study reported that pulp-like tissues were formed by transplanting 3D DPSC constructs into human root canals after subcutaneous implantation into immunodeficient mice [7]. Another study revealed that hDPSCs combined with NF-SMS were used to fill the pulp cavity and form pulp-like tissues similar to native pulp in a rat model [8]. However, true regeneration of DPC has not been achieved in RE [9–14]. To improve the outcomes of RE, a key issue is to improve DPSC function in dental clinic conditions; therefore, it is necessary to explore the mechanisms controlling the directed differentiation of DPSCs in these conditions.

The microenvironmental niche of MSCs, which maintains and regulates their function, is the key element for determining the characteristics of MSCs. Oxygen in the microenvironment is necessary for the normal growth and differentiation of cells. Oxygen tensions in human organs and tissues vary from 2 to 9% in physiological conditions. In pathologic conditions or in some relatively hypoxic areas, oxygen tensions can drop to 1% or, in some cases, lower [15]. Oxygen is required for ATP generation, which is necessary to provide free energy for cell survival. Cells maintain the supply of ATP via oxidative phosphorylation in mitochondria. The survival of cells is threatened with the loss of oxygen. Cells can sense changes in oxygen tension, initiate or inhibit gene transcription via the stimulation of hypoxia-inducible factors, and activate adaptive processes that can enhance cell survival in the case of limited oxygen [16]. The effective isolation, culture, and expansion of MSCs under hypoxic conditions will mimic the physical microenvironmental niche and is crucial for maintaining and protecting implanted MSCs and beneficial for tissue regeneration in the physical hypoxic environment. Currently, MSCs are principally isolated and cultured under normoxia (20–21% O_2_) in vitro, which is markedly different from their typical in vivo environment. Previous studies have indicated that after being cultured in normoxic conditions, dental MSCs recultured in hypoxic conditions possess altered proliferation capacities and differentiation potentials; however, the results are inconclusive due to different cell sources and experimental conditions. Most studies have shown that hypoxia can promote the proliferation of dental stem cells [17–24]. However, some studies have reported that hypoxia had no or negative effects on the proliferation and survival of dental stem cells [25–28]. Some studies have shown that hypoxia can enhance the osteo/odontogenic differentiation of dental stem cells [17, 21, 23, 24, 26, 29–31], while other studies reported the opposite result [18]. In addition, it has been reported that cell death can occur under severe hypoxia (0.01% O_2_) or anoxia [27]. MSC-mediated regenerative endodontics is a promising method for damaged teeth; however, root canals also provide a hypoxic environment for MSCs [32]. There is little known about the effects of hypoxia on dental stem cells and how to improve their function in a hypoxic environment. Thus, there is a need to discover DPSC characteristics and regulatory mechanisms in the hypoxic environment of root canals.

Here, 3% O_2_ tension was used to mimic hypoxia, and the characteristics and gene expression profiles of DPSCs in hypoxia were investigated. In addition, bioinformatic analysis was conducted to predict the interactions of coding and noncoding RNA and identify core regulatory factors of DPSCs in hypoxia. Furthermore, loss-of-function assays were performed to detect the effect of one candidate noncoding RNA, six-twelve leukemia (STL), on DPSCs. These results may help reveal the molecular mechanisms of DPSCs in a hypoxic environment, which may contribute to the identification of the key regulators that are necessary for DPSC-mediated dental tissue regeneration.

## Methods

### DPSC isolation and culture

Human teeth were collected with informed patient consent and based on the protocol approved by Beijing Stomatological Hospital, School of Stomatology, Capital Medical University, (Ethics Committee Agreement, Beijing, Stomatological Hospital Ethics Review No. 2011-02). Human dental pulps were obtained from healthy teeth extracted for impaction or other orthodontic reasons. The tissues from the crown and superior 2/3 root pulp were gently separated, and DPSCs were isolated, cultured, and characterized as previously described [33]. The culture media was changed every 3 days. DPSCs from passages 3–5 were used in further experiments. For hypoxic culture, cells were grown in a hypoxic chamber flushed with 3% O_2_ and 5% CO_2_, with a balance of 92% N_2_ at 37 °C by using the hypoxic incubator (Thermo Scientific™ Heracell VIOS 160i, USA) which can accurately adjust the O_2_ concentration of 1–21%_._

### Cell Counting Kit-8 (CCK8) assay

The proliferation of DPSCs was determined using the CCK8 kit, according to the manufacturer’s instructions (Dojindo Laboratories, Kumamoto, Japan). A volume of 100 μL of DPSC suspension (3000 cells/well) was dispensed into the wells of a 96-well plate. After preincubation for 2 or 3 days in a humidified incubator (37 °C, 5% CO_2_), the culture medium was replaced with 100 μL MEM containing 10 μL CCK8, then incubated for 2 h at 37 °C. Absorbance (OD) at 450 nm was measured in a multiwell spectrophotometer.

### CFSE assay

Carboxyfluorescein succinimidyl ester (CFSE) assays were performed according to the CellTrace™ CFSE Cell Proliferation Kit protocol (Invitrogen, Carlsbad, CA, USA) for labeling cells in suspension as previously described [33]. Briefly, DPSCs were stained, then seeded at a density of 1.0 × 10^5^ cells/plate in six well plates. After 3 days of culture, DPSCs were harvested and analyzed using a flow cytometer (FACSCalibur, BD Biosciences, USA) with 635 nm excitation and emission filter appropriate for far red. The proliferation index was calculated by Modfit LT (Verity Software House, Topsham, ME, USA).

### Alkaline phosphatase (ALP) activity assay and Alizarin red staining

To detect mineralization, DPSCs were grown in mineralization-inducing medium consisting of α-MEM, 15% FBS, 2 mM/L glutamine, 100 U/mL penicillin, 100 μg/mL streptomycin, 100 μM/L ascorbic acid, 10 mM/L β-glycerophosphate (Sigma-Aldrich, St. Louis, MO, USA), 1.8 mM/L of KH_2_PO_4_, and 10 nM/L dexamethasone (Sigma-Aldrich). Cells were induced for 5 days, and an ALP activity assay was performed as previously described [33]. DPSCs were induced for 2 or 3 weeks, and Alizarin red staining and quantitative calcium analysis was performed as previously described [33].

### Real-time RT-PCR

Real-time RT-PCR was used to detect the expression levels of osteo/odontogenic marker genes and to verify the changes in gene expression that were detected by microarray analysis. Total RNA was isolated from DPSCs with TRIzol reagent (Invitrogen). cDNA was synthesized using reverse transcriptase with random primers for mRNA and lncRNA and with a special stem-loop primer for miRNA. The QuantiTect SYBR Green PCR kit (Qiagen, Hilden, Germany) was used in real-time PCR reactions, and the IcycleriQ multicolor real-time PCR detection system was used (Bio-Rad, Hercules, CA, USA). The gene expression levels were evaluated by normalizing the PCR signal to that of GAPDH (mRNA and lncRNA) or U6 (miRNA) and applying the 2^−△△CT^ calculation. Each reaction was run in triplicate, and the entire procedure was repeated three times. The primers were designed by applying the online D-LUX DesignerTM program (Invitrogen) and are shown in Additional file 1: Table S1.

### Western blot analysis

To detect proteins of osteo/odontogenic differentiation genes, DPSCs were cultured in mineralization-inducing medium for 2 weeks, and then western blot analysis was performed as previously described [34]. Primary antibodies were obtained from commercial sources as follows: polyclonal antibody against bone sialoprotein (BSP) (Cat. No. bs-2668R, Bioss, Beijing, China); monoclonal antibody against osteocalcin (OCN) (Cat. No. ab133612, Abcam, Cambridge, UK); polyclonal antibody against dentin sialophosphoprotein (DSPP) (Cat. No. bs-10316R Bioss, Beijing, China); polyclonal antibody against beta-actin (β-actin) (Cat. No. AC026, Abclonal, Wuhan, China).

### RNA preparation and microarray analysis

To detect differential coding and noncoding transcript expression profiles, DPSCs from three different individuals were cultured in normoxia or hypoxia for 3 days. Total RNA was then extracted from DPSCs using TRIzol and the RNeasy mini kit (Qiagen). The quality and quantity of RNA were verified by multiImager and spectrophotometer (Meriton, Beijing, China) analysis. The RNA labeling, hybridization, and scanning of the chips (Affymetrix HTA2.0 Array for mRNA and lncRNA, Affymetrix GeneChip miRNA 4.0 Array for miRNA) were performed as outlined in the Affymetrix technical manual by Cnkingbio Corp (Beijing, China). The threshold set for up- or downregulated mRNA, lncRNA, or miRNA was a fold change > 1.5 and a *p* value < 0.05.

### Bioinformatic analysis

The microarray raw data were normalized for follow-up analysis with Expression Console software (Affymetrix, CA, USA) using the MAS 5.0 statistical algorithm. The bioinformatic analysis was performed after normalized data was compared and filtered. Hierarchical clusters were performed by EPCLUST. Gene ontology (GO) analysis was used to analyze the genes’ primary functions. Pathway analysis was used to determine the significantly changed pathways of the differential genes based on KEGG, Biocarta, and Reatome. A coding-noncoding gene coexpression (CNC) network and miRNA target gene network were constructed to identify the interactions among genes and to locate core regulatory factors that played an important role in these networks.

### Viral infection

STL shRNA (STLsh) and control shRNA (Consh) were purchased from Genepharma (Suzhou, China). The lentiviruses were transfected into DPSCs in the presence of polybrene (6 μg/mL, Sigma) for 12 h. After 48 h, the transfected DPSCs were selected with 1 μg/mL puromycin for 3 days. The target sequences for the shRNA were STLsh, 5′-CGGCATGACTAAGAGATATCG-3′ and Consh, 5′-TTCTCCGAACGTGTCACGT-3′.

### Oil Red O staining

To detect adipogenic differentiation, DPSCs were grown in adipogenic-inducing medium for 3 weeks under normoxic conditions, and then Oil Red O staining was performed as previously described [33]. The OD at 500 nm was measured using 100% isopropanol as a blank.

### Statistical analysis

The statistical analyses were performed by using SPSS 16.0 software (SPSS Inc., Chicago, IL, USA). Significance was determined by the Student’s *t* test or one-way ANOVA analysis. For all tests, *p* < 0.05 was considered statistically significant.

## Results

### Hypoxia enhanced the proliferation ability and inhibited the osteo/odontogenic differentiation potential of DPSCs

**Fig. 1:**
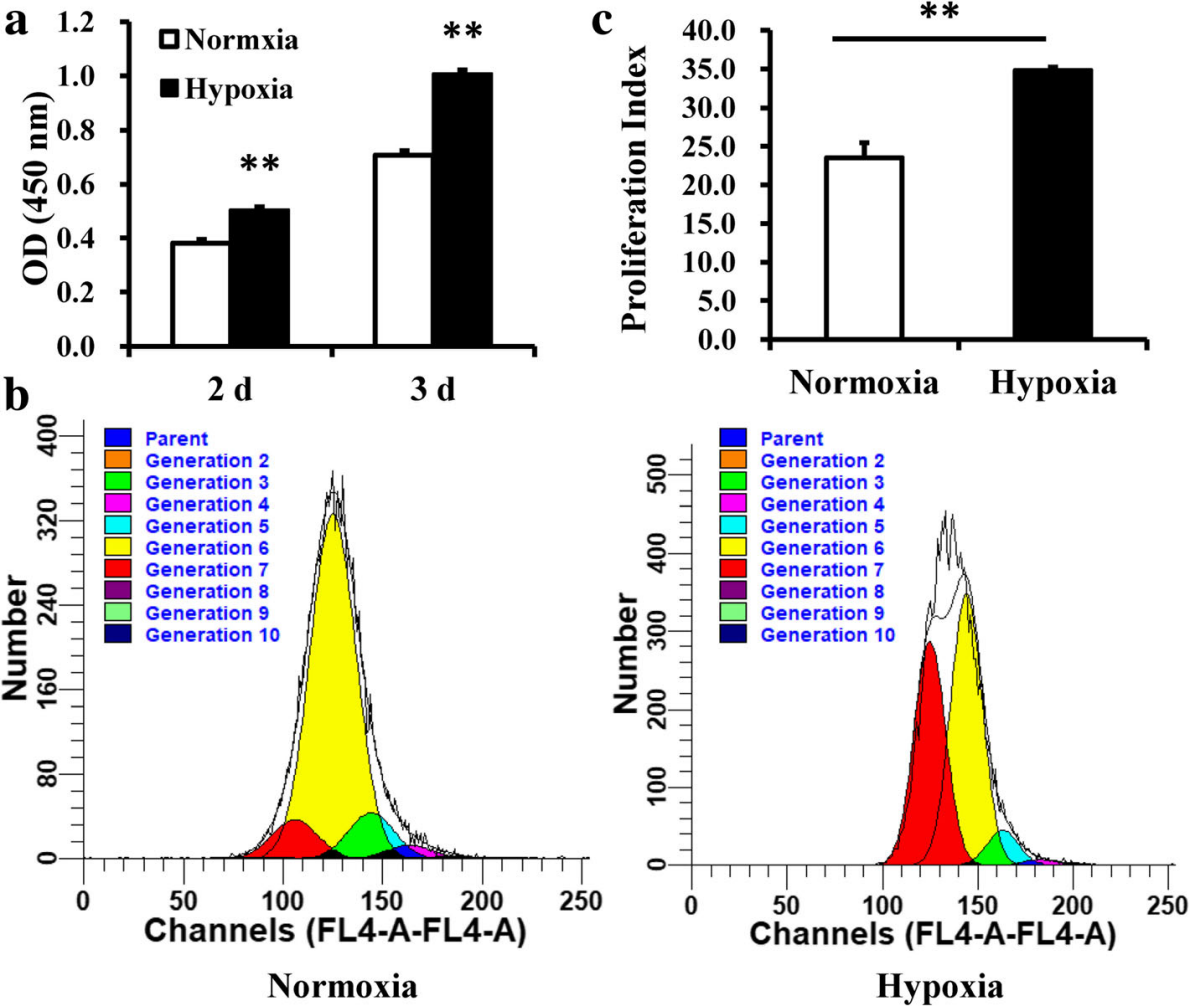
Hypoxia promoted cell proliferation in DPSCs. **a** CCK8 assays revealed that hypoxia resulted in increased OD values at days 2 and 3. **b**, **c** CFSE assays showed that hypoxia resulted in increased cell proliferation at day 3. Student’s *t* tests were performed to determine statistical significance. All error bars represent SD (*n* = 3). **p < 0.01

**Fig. 2:**
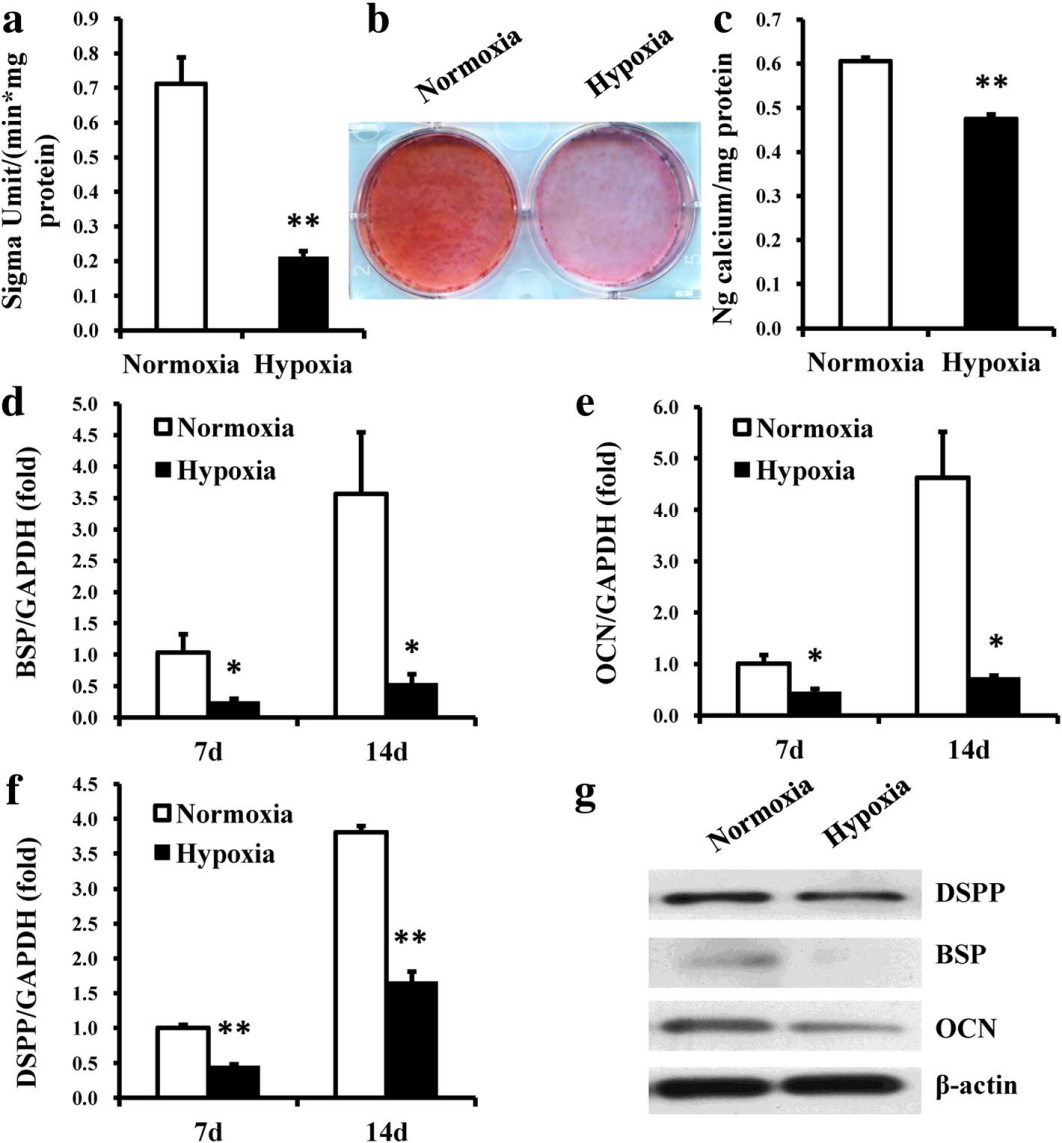
Hypoxia inhibited osteo/odontogenic differentiation in DPSCs. **a** ALP activity of DPSCs was reduced in the hypoxia group 5 days after osteo/odontogenic induction. **b**, **c** Alizarin red staining (**b**) and calcium quantitation (**c**) showed a significant decrease in mineralization of DPSCs in the hypoxia group 2 weeks after osteo/odontogenic induction. **d–f** Real-time RT-PCR indicated that the expression levels of BSP, OCN, and DSPP in DPSCs in hypoxic conditions were inhibited 7 and 14 days after osteo/odontogenic induction. **g** Western blot results showed that hypoxia inhibited the expressions of BSP, OCN, and DSPP at 2 weeks after osteo/odontogenic induction. GAPDH was used as an internal control for real-time RT-PCR, and β-actin was used as an internal control for western blot analysis. Student’s *t* tests were performed to determine statistical significance. All error bars represent SD (*n* = 3). *p < 0.05, **p < 0.01

To investigate the effects of hypoxia on DPSCs, DPSCs were cultured in both normoxic and hypoxic conditions. CCK8 assays showed that the cell numbers of DPSCs were increased in hypoxic conditions compared with normoxic conditions at days 2 and 3 (Fig. 1a). CFSE assays also confirmed that the cell proliferation of DPSCs was increased in the hypoxia group compared with the normoxia group at day 3 (Fig. 1b, c). We then tested whether hypoxia could affect the osteo/odontogenic differentiation abilities of DPSCs. DPSCs were cultured in mineralization-inducing medium in both normoxic and hypoxic conditions. ALP activity, an early indicator for osteo/odontogenic differentiation in DPSCs, was determined at 5 days postinduction. The results showed that hypoxia resulted in a significant decrease in the ALP activity of DPSCs (Fig. 2a). After 2 weeks of induction, Alizarin red staining and calcium quantitation revealed weakened mineralization of DPSCs in hypoxic conditions compared with the control group (Fig. 2b, c). Real-time RT-PCR showed that the osteo/odontogenic marker genes BSP, OCN, and DSPP were downregulated at 7 and 14 days postinduction in the hypoxia group compared with the normoxia group (Fig. 2d–f). Western blot results also showed that the expressions of BSP, OCN, and DSPP in DPSCs were downregulated at 2 weeks postinduction in the hypoxia group (Fig. 2g).

### The profiling of differentially expressed mRNAs, lncRNAs, and miRNAs in DPSCs under hypoxic and normoxic conditions

**Fig. 3:**
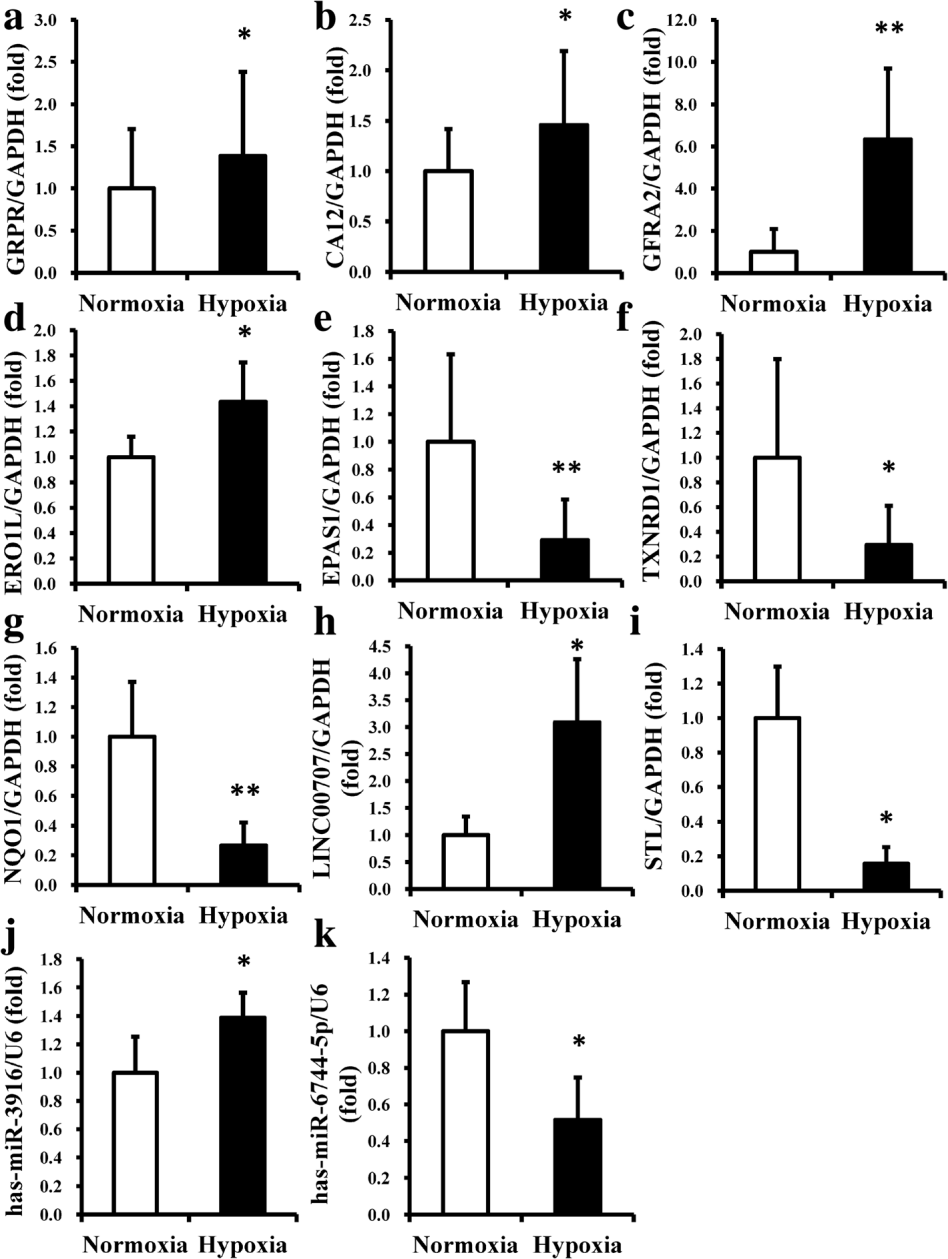
The expression levels of selected mRNAs, lncRNAs, and miRNAs of DPSCs by real-time RT-PCR. **a**–**d** mRNA expression levels of GRPR, CA12, GFRA2, and ERO1L were increased in the hypoxia group. **e**–**g** mRNA expression levels of EPAS1, TXNRD1, and NQO1 showed decreased expression in the hypoxia group. **h** The lncRNA LINC00707 showed increased expression in the hypoxia group. **i** The lncRNA STL showed decreased expression in the hypoxia group. **j** The miRNA hsa-miR-3916 showed increased expression in the hypoxia group. **k** The miRNA hsa-miR-6744-5p showed decreased expression in the hypoxia group. GAPDH was used as an internal control for mRNA and lncRNA, and U6 was used as an internal control for miRNA. Student’s *t* tests were performed to determine statistical significance. All error bars represent SD (*n* = 5). *p < 0.05, **p < 0.01

To illuminate why hypoxia could affect the proliferation capacity and differentiation potential of DPSCs, microarray and bioinformatic analyses were performed to identify and locate core regulators of DPSCs in hypoxia. The RVM *t* test was applied to filter the genes that were differentially expressed, and the differentially expressed genes with 1.5-fold changes and *p* values < 0.05 were selected for subsequent analysis. From the microarray data, a total of 60 mRNAs with differential expression in DPSCs were identified between the hypoxia and normoxia groups, of which 25 were upregulated and 35 were downregulated in the hypoxia group compared with those in the normoxia group (Additional file 2: Table S2). A total of 47 lncRNAs in DPSCs were significantly differentially expressed between the hypoxia and normoxia groups, of which 20 were upregulated and 27 were downregulated in the hypoxia group compared with those in the normoxia group (Additional file 3: Table S3). In addition, a total of 14 miRNAs in DPSCs were differentially expressed between the hypoxia and normoxia groups, of which seven were upregulated and seven were downregulated in the hypoxia group compared with the normoxia group (Additional file 4: Table S4). To confirm the reliability of the microarray data, seven differentially expressed mRNAs (GRPR, CA12, GFRA2, ERO1L, EPAS1, TXNRD1, and NQO1), two differentially expressed lncRNAs (LINC00707 and STL), and two differentially expressed miRNAs (hsa-miR-3916 and hsa-miR-6744-5p) were selected, and real-time RT-PCR was performed to detect their expression levels. The results showed that the expression levels of the selected coding and noncoding RNAs were consistent with the microarray results and confirmed the reliability of the microarray data (Fig. 3).

GO analysis was performed to determine the significantly changed functions obtained from the differentially expressed mRNAs of DPSCs in hypoxic conditions compared with normoxic conditions. A total of 91 GO functions (*p* < 0.01, FDR < 0.05) were obtained, of which 28 were upregulated and 63 were downregulated in the hypoxia group. The negative logarithm of the *p* value (− LgP) was applied to show the correlation of gene expression levels and the relevant biological processes (Additional file 5: Figure S1 and Additional file 6: Figure S2). The GO functions enriched from the upregulated genes were related to glycogen biosynthetic process, positive regulation of DNA metabolic process, wound healing, negative regulation of mesoderm development, negative regulation of mitotic sister chromatid separation, cellular protein modification process, and several others. The GO functions enriched from downregulated genes included mesoderm formation, positive regulation of glutamate-cysteine ligase activity, response to hypoxia, oxidation-reduction process, pentose biosynthetic process, regulation of cell growth, and several others.

Pathway analysis using the KEGG database was then performed to enrich for the major pathways that were significantly associated with the differentially expressed mRNAs of DPSCs in hypoxia compared with the normoxia group. A total of 17 pathways (*p* < 0.05) were identified, among which 5 were upregulated and 12 were downregulated in the hypoxia group (Additional file 7: Figure S3 and Additional file 8: Figure S4). The upregulated pathways were starch and sucrose metabolism, insulin resistance, neurotrophin signaling pathway, oocyte meiosis, and nitrogen metabolism. The downregulated pathways included Wnt signaling pathway, pentose phosphate pathway, glutathione metabolism, ubiquinone and other terpenoid-quinone biosynthesis, hypoxia-inducible factor-1 (HIF-1) signaling pathway, carbon metabolism, and several others.

**Fig. 4:**
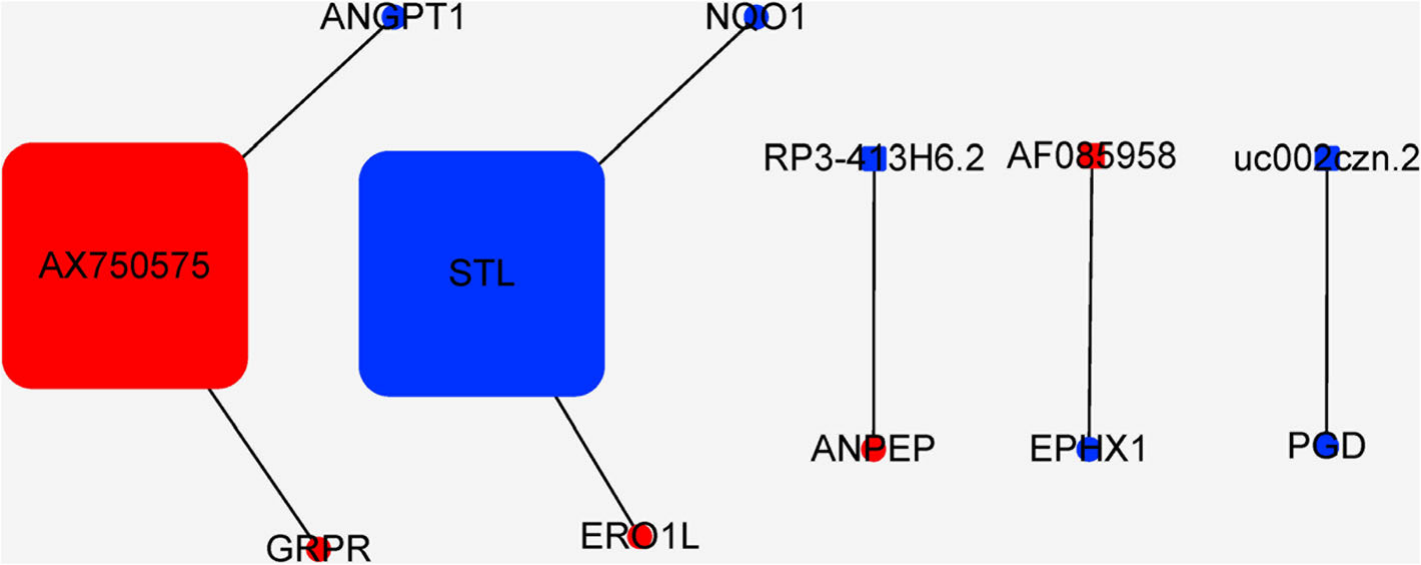
The coexpression network (CNC) of mRNAs and lncRNAs of DPSCs in hypoxia. Circles represent upregulated (red) and downregulated (blue) mRNAs in DPSCs in hypoxia. Squares represent upregulated (red) and downregulated (blue) lncRNAs in DPSCs in hypoxia. Lines indicate the regulatory relationships between mRNAs and lncRNAs. The size of each circle and square represents the degree of centrality

**Fig. 5:**
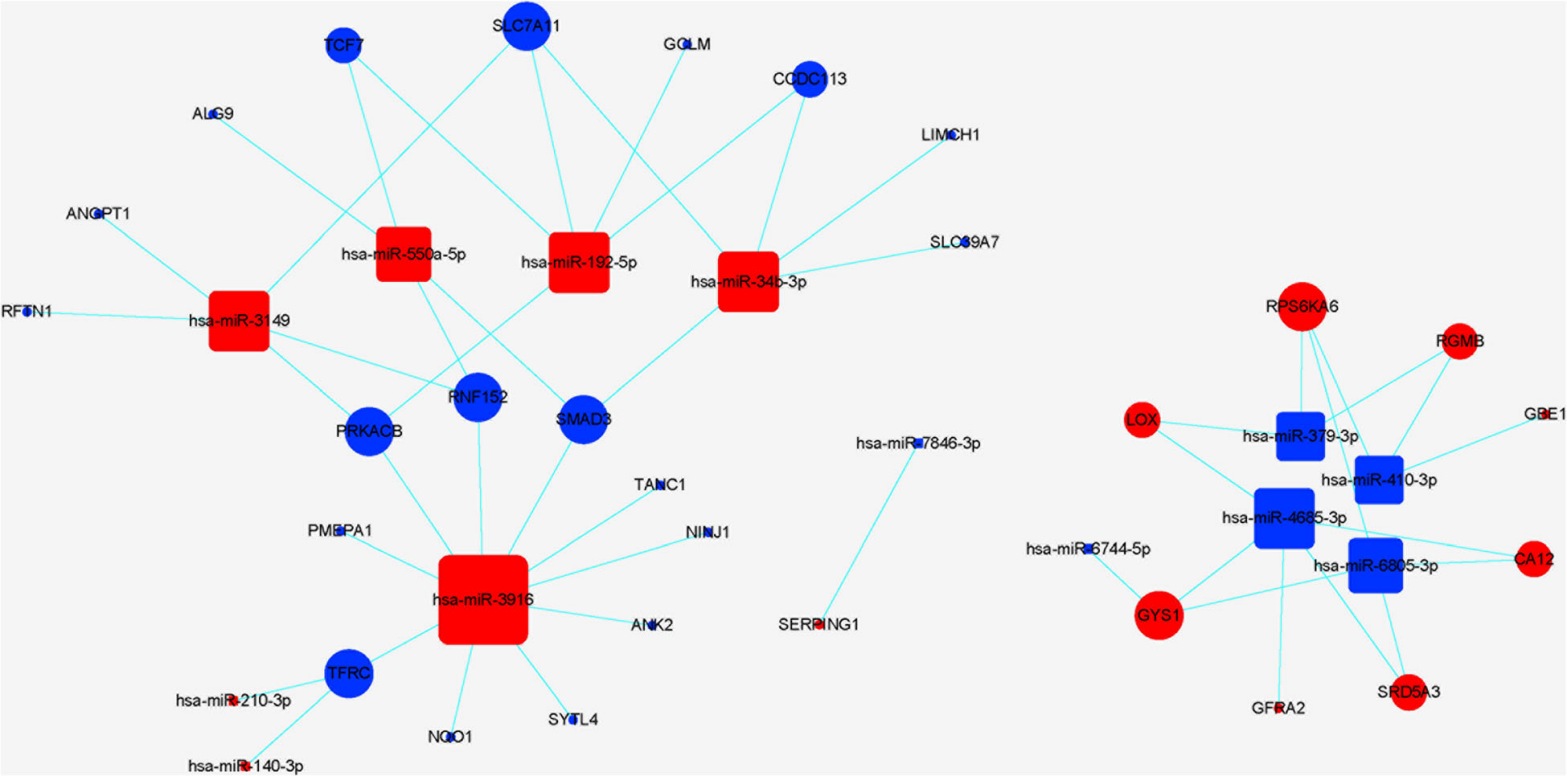
The miRNA target gene network of DPSCs in hypoxia. Circles represent upregulated (red) and downregulated (blue) mRNAs in DPSCs in hypoxia. Squares represent upregulated (red) and downregulated (blue) miRNAs in DPSCs in hypoxia condition. Lines indicate the regulatory relationships between mRNAs and miRNAs. The size of each circle and square represents the degree of centrality

Next, a CNC network was constructed to identify the interactions and significance degrees among the differentially expressed mRNAs and lncRNAs based on clustering coefficients and degrees (Fig. 4). Twelve core regulatory genes were identified including seven mRNAs (GRPR, ERO1L, ANPEP, EPHX1, PGD, ANGPT1, and NQO1) and five lncRNAs (AF085958, AX750575, uc002czn.2, RP3-413H6.2, and STL). In addition, a miRNA target gene network was constructed to identify the interactions and significance degrees among the differentially expressed miRNAs and mRNAs (Fig. 5). A total of 41 core regulatory genes were identified including 28 mRNAs (GYS1, PRKACB, NQO1, and several others) and 13 miRNAs (hsa-miR-3916, hsa-miR-192-5p, and several others).

### Depletion of STL inhibited the osteo/odontogenic differentiation potential of DPSCs

**Fig. 6:**
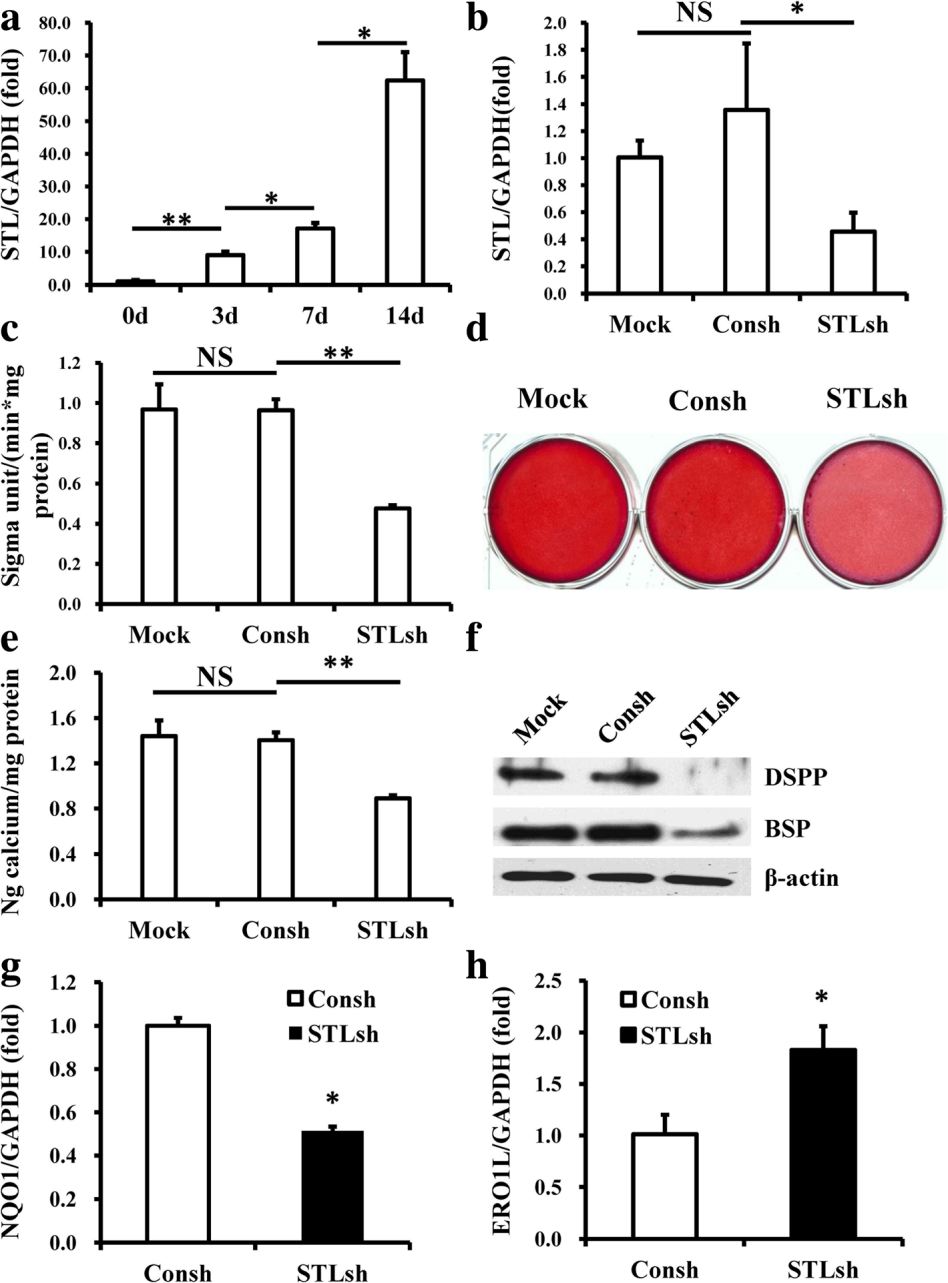
Knockdown of the lncRNA STL inhibited the osteo/odontogenic differentiation potential of DPSCs in vitro. **a** STL expression increased during osteo/odontogenic differentiation of DPSCs evaluated by real-time RT-PCR. **b** Real-time RT-PCR showed knockdown efficacy of STL. **c** ALP activity assay showed that STL depletion reduced ALP activity. **d**, **e** STL depletion repressed the mineralization of DPSCs shown by Alizarin red staining (**d**) and calcium quantitative analysis (**e**). **f** Western blot results showed that knockdown of STL inhibited the expressions of BSP and DSPP in DPSCs after osteo/odontogenic induction. **g**, **h** STL depletion inhibited NQO1 expression (**g**) but promoted ERO1L expression in DPSCs (**h**). GAPDH was used as an internal control for real-time RT-PCR, and β-actin was used as an internal control for western blot analysis. One-way ANOVA and Student’s *t* tests were performed to determine statistical significance. All error bars represent SD (*n* = 3). NS; no significant difference, *p < 0.05, **p < 0.01

Then, we selected one candidate lncRNA, STL, which was identified as a possible core regulatory factor of DPSCs according to the CNC network, to investigate its function in DPSCs. The real-time RT-PCR results showed that the expression of STL increased with the osteo/odontogenic differentiation of DPSCs (Fig. 6a). To investigate the possible regulatory function of STL on DPSCs, STL in DPSCs was knocked down through STL lentiviral shRNA infection. Approximately 70% knockdown efficiency was achieved as determined by real-time RT-PCR (Fig. 6b). To test whether STL could affect the differentiation potential of DPSCs, DPSCs were cultured in osteogenic-inducing medium under normoxic conditions. ALP activity was determined 5 days postinduction, and the results showed a significant decrease in ALP activity after knockdown of STL compared with the Consh and Mock groups (Fig. 6c). After 2 weeks of induction, Alizarin red staining and calcium quantitation revealed weakened mineralization in vitro in STL-depleted DPSCs compared to the Consh and Mock groups (Fig. 6d, e). Western blot results showed that the expressions of BSP and DSPP were downregulated at 2 weeks postinduction in STL-depleted DPSCs compared to the Consh and Mock groups (Fig. 6f). Real-time RT-PCR showed that NQO1 was downregulated and ERO1L was upregulated in STL-depleted DPSCs compared to the control group (Fig. 6g, h). Interestingly, we also found a significant decrease of lipid deposits in STL-depleted DPSCs compared to the Consh and Mock groups at 3 weeks after adipogenic induction (Additional file 9: Figure S5); these indicated that depletion of STL decreased the adipogenic differentiation in DPSCs.

## Discussion

In tissue engineering, MSCs need to be implanted into the tissue of the host after in vitro amplification. During this process, changes in oxygen tension in the extracellular environment can affect the growth and differentiation of MSCs. In the present study, we found that DPSCs exhibited significantly higher proliferation under 3% oxygen. Our results are consistent with those of previous studies, which demonstrated that culturing under appropriate hypoxic conditions or pretreatment with 1–5% oxygen could enhance the proliferation potential of DPSCs [17–20]. In the present study, we also found that hypoxia reduced ALP activity, weakened the mineralization ability of DPSCs, and decreased the expression of BSP, OCN, and DSPP, indicating that the osteo/odontogenic differentiation of DPSCs was inhibited in 3% oxygen. Our results are in agreement with those of a previous study that found ALP activity and the expression of DMP1, DSPP, and OCN were suppressed when DPCs were cultured under hypoxic conditions [18]. However, other studies showed that hypoxia could promote the mineralization of DPSCs and upregulate mineral-associated genes [17, 21, 29]. These different results may be caused by different oxygen tensions and action times. Our results confirm that oxygen tension is an important regulator and suggest that 3% oxygen promotes cell proliferation and inhibits DPSC differentiation.

For successful cell therapy, the molecular mechanisms that underlie the effects of hypoxia on human DPSCs need to be elucidated. Therefore, the expression profiles of mRNAs, lncRNAs, and miRNAs of DPSCs (in hypoxia vs normoxia) were determined, and the reliability of the microarray data was confirmed by real-time RT-PCR. In total, 60 differentially expressed mRNAs, 47 differentially expressed lncRNAs, and 14 differentially expressed miRNAs were identified. These differentially expressed coding and noncoding RNAs may be candidates for key regulators controlling the proliferation and differentiation of DPSCs in hypoxia and warrant further bioinformatic analysis.

By GO and pathway analysis, we screened out the significantly altered functions and pathways associated with DPSCs in hypoxia, including the Wnt and HIF-1 signaling pathways. The canonical Wnt/β-catenin signaling pathway has different functions in hard tissue development and regeneration, depending on cell type and stage of development. Previous reports found that the proliferation and differentiation potentials of DPSCs could be regulated via the Wnt/β-catenin signaling pathway [35, 36]. HIF-1, which consists of two subunits, HIF-1α and HIF-1β, is known as a key nuclear transcription factor related to hypoxia. HIF-1α is regulated by the cellular oxygen concentration and determines the transcriptional activity of HIF-1. HIF-1α can play an important role in regulating the proliferation, differentiation, and pluripotency of stem cells through signaling pathways such as Wnt, Notch, and Sonic hedgehog (Shh) [25, 37]. It was reported that HIF-1α protects MSCs via the induction of autophagy and the PI3K/AKT/mTOR pathway [38]. A study in porcine dental pulp-derived cells under severe hypoxic conditions reported that the expression of BSP, DMP-1, and DSPP was repressed, while the expression of HIF-1α was increased at 6 and 12 h then downregulated after 18 h [27]. From these data, we speculate that the Wnt and HIF-1 signaling pathways could contribute to DPSC characteristics in hypoxic conditions.

According to CNC network and miRNA target gene network analysis, some of the differentially expressed RNAs in the two groups were screened out as possible regulatory factors of DPSCs in hypoxia, including ERO1L, NQO1, STL, hsa-miR-192-5p, and several others. NQO1 can function as a direct superoxide anion radical scavenger or a major target of the Nrf2 pathway to maintain cellular redox homeostasis and attenuate cellular oxidative stress. In addition to oxidation-reduction process, NQO1 is related to obesity, hypertension, renal injury, bone metabolism, tumor growth, and other processes [39–41]. It was reported that NQO1 and HO-1 were significantly downregulated in human primary endothelial cells that had been adapted to physiological O_2_ (5%) according to microarray analysis and further validation, which is consistent with our results [42]. NQO1 may act downstream of the Nrf2 pathway, and it was revealed that the mineralization of human periodontal ligament fibroblasts was improved by downregulating Nrf2 through activation of the Wnt/β-catenin pathway [43]. ERO1L belongs to the family of classic oxygen-regulated genes, and its expression is augmented under hypoxia, which is consistent with our results [44]. It was revealed that the expression of ERO1L was regulated via the HIF pathway during hypoxia [44, 45]. ERO1L controls several proteins via reoxidation of “client” protein disulfide isomerase. Furthermore, it can regulate cell proliferation and plays crucial roles in the progression of various tumors, cardiovascular diseases, diabetes, and other diseases [46–51]. As a miRNA, miR-192-5p plays an important role in cell proliferation and differentiation [52–54]. Emerging evidence has further suggested its role in the development of tumors, cardiovascular diseases, liver disease, and other diseases [55–57]. Consistent with our results, it has been reported that the release of miR-192-5p may be attributed to oxidative stress, and its expression was regulated by hypoxia [58, 59]. As an lncRNA, STL does not contain any long open reading frames. Emerging evidence revealed that lncRNAs play important roles in the proliferation and osteogenic differentiation capacity of MSCs through different regulatory mechanisms, including transcription factor binding and chromatin modification [60–64]. Some lncRNAs have been identified as key regulators of the proliferation capacity and differentiation potential of DPSCs [33, 65]. It has also been reported that STL was related to hematologic malignancies and uterine tumors resembling ovarian sex cord tumors [66–68]. According to the CNC network, STL showed positive and negative regulatory effects on NQO1 and ERO1L, respectively. Due to the effects of NQO1 and ERO1L on the functions of stem cells, we speculate that STL may be a key regulator of these processes.

Indeed, we found that the expression of STL in DPSCs was enhanced along with osteo/odontogenic differentiation of DPSCs, indicating that STL may play a positive role in this process. We therefore conducted loss-of-function assays to investigate the functions of STL in DPSCs. Our results showed that the knockdown of STL inhibited the osteo/dentinogenic differentiation potential of DPSCs, indicating that STL was involved in the positive regulation of osteo/dentinogenic differentiation ability of DPSCs. Consistent with the CNC network, NQO1 was downregulated while ERO1L was upregulated when STL was knocked down in DPSCs. In addition, STL and NQO1 were downregulated and ERO1L was upregulated in hypoxia, indicating that decreased STL might be one factor for the impaired osteo/dentinogenic differentiation ability of DPSCs in hypoxia and that this process might be downregulated via NQO1 and upregulated via ERO1L. It is possible that STL works on DPSCs via interactions with these two genes, and therefore, STL may be considered as a possible therapeutic target for dental tissue regeneration. However, further studies are needed to verify this hypothesis and the underlying mechanism.

## Conclusions

In conclusion, our results revealed that 3% oxygen tension could enhance the proliferative ability and impair the osteo/odontogenic differentiation potential of DPSCs, indicating that hypoxia is an important factor affecting DPSC-mediated dental tissue regeneration. Furthermore, our results identified that candidate coding and noncoding RNA could be potential targets for improving the DPSC characteristics in hypoxia and lead to a better understanding of the mechanisms of hypoxia’s effects on DPSCs. In addition, our results highlighted the significant involvement of one lncRNA, STL, in the positive regulation of the osteo/odontogenic differentiation ability of DPSCs and indicated that STL could be a potential target in regenerative endodontics.

## Additional files

Additional file 1: **Table S1.** Primer sequences used in real-time RT-PCR analysis. (DOCX 16 kb)

Additional file 2: **Table S2.** The differentially expressed mRNAs of hDPSCs in hypoxic and normoxic conditions. (DOCX 18 kb)

Additional file 3: **Table S3.** The differentially expressed lncRNAs of hDPSCs in hypoxic and normoxic conditions. (DOCX 20 kb)

Additional file 4: **Table S4.** The differentially expressed miRNAs of hDPSCs in hypoxic and normoxic conditions. (DOCX 14 kb)

Additional file 5: **Figure S1.** Significant GOs of upregulated genes of DPSCs in hypoxia. The *x* axis shows − LgP and the y axis shows GO category. A larger – LgP indicates a smaller *p* value for the difference. (TIFF 1845 kb)

Additional file 6: **Figure S2.** Significant GOs of downregulated genes of DPSCs in hypoxia. The *x* axis shows − LgP and the y axis shows GO category. A larger − LgP indicates a smaller *p* value for the difference. (TIFF 2370 kb)

Additional file 7: **Figure S3.** Significant pathways of upregulated genes of DPSCs in hypoxia. The x axis shows − LgP and the y axis shows pathways category. A larger – LgP indicates a smaller *p* value for the difference. (TIFF 512 kb)

Additional file 8: **Figure S4.** Significant pathways of downregulated genes of DPSCs in hypoxia. The *x* axis shows − LgP and the y axis shows pathways category. A larger – LgP indicates a smaller *p* value for the difference. (TIFF 1140 kb)

Additional file 9: **Figure S5.** Knockdown of STL inhibited the adipogenic differentiation of DPSCs in vitro. (a, b) Oil red staining (a) and a quantitation analysis (b) revealed a significant decrease in lipid deposits in STL-depleted DPSCs compared to the control groups 3 weeks after adipogenic induction. One-way ANOVA was performed to determine statistical significance. All error bars represent SD (*n* = 3). NS; no significant difference, **p < 0.01. Scale bar 200 μm. (TIFF 1495 kb)

## Abbreviations

ALP: Alkaline phosphatase
BSP: Bone sialoprotein
CFSE: Carboxyfluorescein succinimidyl ester
CNC: Coding-noncoding gene coexpression
DPSCs: Dental pulp stem cells
DSPP: Dentin sialophosphoprotein
ERO1L: Endoplasmic reticulum oxidoreductase 1-like
GO: Gene ontology
HIF-1: Hypoxia-inducible factor-1
KEGG: Kyoto Encyclopedia of Genes and Genomes
LncRNA: Long noncoding RNA
MiRNA: MicroRNA
MSCs: Mesenchymal stem cells
NQO1: NAD(P)H dehydrogenase quinone 1
OCN: Osteocalcin
RT-PCR: Reverse transcription-polymerase chain reaction
shRNA: Short-hairpin RNA
STL: Six-twelve leukemia
WNT: Wingless-type MMTV integration site family
