# Supplementary material for: Analysis of the characteristics and expression profiles of coding and noncoding RNAs of human dental pulp stem cells in hypoxic conditions
Source: Stem Cell Res Ther. 2019 Mar 12;10:89. doi: 10.1186/s13287-019-1192-2 (PMC6417198; doi:10.1186/s13287-019-1192-2)
Supplement: Supplementary file 1 — Table S1. Primer sequences used in real-time RT-PCR analysis. (DOCX 16 kb) [file 13287_2019_1192_MOESM1_ESM.docx]

| **Table S1.** Primers sequences used in real-time RT-PCR analysis | |
| --- | --- |
| **Gene symbol** | **Primer sequence (5’-3’)** |
| GAPDH-F | CGAACCTCTCTGCTCCTCCTGTTCG |
| GAPDH-R | CATGGTGTCTGAGCGATGTGG |
| DSPP-F | CGACATAGGTCACAATGAGGATGTCG |
| DSPP-R | TTGCTTCCAGCTACTTGAGGTC |
| BSP-F | CAGGCCACGATATTATCTTTACA |
| BSP-R | CTCCTCTTCTTCCTCCTCCTC |
| OCN-F | GCGCTACCTGTATCAATGGC |
| OCN-R | AACTCGTCACAGTCCGGATT |
| GRPR-F | ATGGCTATTTGGCAGGATTG |
| GRPR-R | GCCGTGAGTGTGAAGACAGA |
| CA12-F | CTCCTTCTCCCAAGTGCAAG |
| CA12-R | AAAAGCCAAATGGACACCAC |
| GFRA2-F | CCCAGGGAGTAACAAGGTG |
| GFRA2-R | CAGCCTACAAGGCCAGTTTC |
| NQO1-F | CTGGTTTGAGCGAGTGTTCA |
| NQO1-R | CGGAAGGGTCCTTTGTCATA |
| TXNRD1-F | TAAGGAGGCAGCCCAATATG |
| TXNRD1-R | ACACATGTTCCTCCGAGACC |
| EPAS1-F | GGGCCAGGTGAAAGTCTACA |
| EPAS1-R | TGCTGGATTGGTTCACACAT |
| ERO1L-F | ATCCTGAGCGCTACACTGGT |
| ERO1L-R | CTTGTCCCTTGACCAGAAGC |
| STL-F | TTCCCAGAACGGAAAATCAG |
| STL-R | TCTCCGCATCTTTGTCTGTG |
| LINC00707-F | TCGGCCCATTTCTCACTAGC |
| LINC00707-R | ACGGTGGCAGTATGGTGAAT |
| U6-F | CGCTTCGGCAGCACATATAC |
| U6-R | ATGGAACGCTTCACGAATTTG |
| hsa-miR-3916-F | AAGAGGAAGAAATGGCTGGTTC |
| hsa-miR-6744-5p-F | ATTGGATGACAGTGGAGGCC |
| hsa-miR-TY-R | TGCGTGTCGTGGAGTCG |
