# Supplementary material for: Analysis of the characteristics and expression profiles of coding and noncoding RNAs of human dental pulp stem cells in hypoxic conditions
Source: Stem Cell Res Ther. 2019 Mar 12;10:89. doi: 10.1186/s13287-019-1192-2 (PMC6417198; doi:10.1186/s13287-019-1192-2)
Supplement: Supplementary file 2 — Table S2. The differentially expressed mRNAs of hDPSCs in hypoxic and normoxic conditions. (DOCX 18 kb) [file 13287_2019_1192_MOESM2_ESM.docx]

| **Table S2.** The differentially expressed mRNAs of hDPSCs in hypoxic and normoxic conditions | | | | |
| --- | --- | --- | --- | --- |
| **Gene Symbol** | **Genbank Accession** | **Fold Change  ( hypoxia/ normoxia)** | **Regulation** | ***p*-value** |
| GRPR | NM_005314 | 4.50 | up | 0.042 |
| CA12 | NM_001218 | 3.49 | up | 0.005 |
| SERPING1 | M13203 | 3.22 | up | 0.031 |
| GFRA2 | NM_001165038 | 2.46 | up | 0.020 |
| SDC4 | NM_002999 | 2.05 | up | 0.046 |
| ERO1L | NM_014584 | 1.91 | up | 0.003 |
| SRD5A3 | NM_024592 | 1.84 | up | 0.004 |
| OR10AG1 | NM_001005491 | 1.84 | up | 0.025 |
| ANXA3 | NM_005139 | 1.83 | up | 0.041 |
| PRPF40B | ENST00000508736 | 1.78 | up | 0.022 |
| LOX | NM_001178102 | 1.73 | up | 0.003 |
| S100A10 | CR542162 | 1.70 | up | 0.043 |
| TMEM45A | NM_018004 | 1.68 | up | 0.020 |
| ANPEP | NM_001150 | 1.62 | up | 0.005 |
| DOCK2 | NM_004946 | 1.55 | up | 0.001 |
| RGMB | NM_001012761 | 1.55 | up | 0.010 |
| GBE1 | NM_000158 | 1.55 | up | 0.016 |
| GYS1 | NR_027763 | 1.55 | up | 0.048 |
| C4orf3 | NM_001001701 | 1.52 | up | 0.010 |
| TRAV41 | ENST00000390468 | 1.51 | up | 0.019 |
| PTTG2 | NM_006607 | 1.50 | up | 0.004 |
| NGF | NM_002506 | 1.50 | up | 0.016 |
| IGKV6D-41 | ENST00000390271 | 1.50 | up | 0.031 |
| ZNF66 | ENST00000360204 | 1.50 | up | 0.034 |
| RPS6KA6 | NM_014496 | 1.50 | up | 0.038 |
| TCF7 | NR_033449 | -1.50 | down | 0.008 |
| SMAD3 | EU016553 | -1.51 | down | 0.000 |
| RNF152 | BC071787 | -1.51 | down | 0.010 |
| PRKACB | AY927367 | -1.51 | down | 0.021 |
| CCDC113 | NM_001142302 | -1.52 | down | 0.009 |
| TANC1 | NM_001145909 | -1.53 | down | 0.011 |
| WARS | NM_004184 | -1.53 | down | 0.025 |
| SLC39A7 | NM_001077516 | -1.54 | down | 0.008 |
| LACC1 | NM_001128303 | -1.55 | down | 0.002 |
| FRAT2 | NM_012083 | -1.56 | down | 0.014 |
| PDCD2 | AK094428 | -1.57 | down | 0.023 |
| H2AFY2 | NM_018649 | -1.59 | down | 0.030 |
| PMEPA1 | AJ308021 | -1.62 | down | 0.031 |
| ANK2 | NM_001127493 | -1.63 | down | 0.013 |
| PIR | NM_001018109 | -1.65 | down | 0.038 |
| GCLM | NM_002061 | -1.68 | down | 0.042 |
| RFTN1 | NM_015150 | -1.71 | down | 0.038 |
| NINJ1 | NM_004148 | -1.72 | down | 0.022 |
| SYTL4 | AK098473 | -1.76 | down | 0.006 |
| FRY | NM_023037 | -1.79 | down | 0.009 |
| ALG9 | AK025214 | -1.83 | down | 0.024 |
| TALDO1 | NM_006755 | -1.85 | down | 0.015 |
| GATSL2 | NM_001145064 | -1.90 | down | 0.034 |
| DHRS3 | DQ426869 | -2.01 | down | 0.020 |
| GATSL1 | NM_001145063 | -2.03 | down | 0.038 |
| LIMCH1 | NM_001112717 | -2.05 | down | 0.003 |
| EPHX1 | NM_000120 | -2.43 | down | 0.020 |
| PGD | NM_002631 | -2.50 | down | 0.027 |
| ANGPT1 | NM_001146 | -2.52 | down | 0.012 |
| TFRC | NM_001128148 | -2.71 | down | 0.035 |
| RGS2 | NM_002923 | -2.80 | down | 0.007 |
| EPAS1 | NM_001430 | -2.98 | down | 0.011 |
| TXNRD1 | NM_001093771 | -3.02 | down | 0.024 |
| NQO1 | NM_000903 | -3.28 | down | 0.005 |
| SLC7A11 | NM_014331 | -4.39 | down | 0.044 |
