# Supplementary material for: Analysis of the characteristics and expression profiles of coding and noncoding RNAs of human dental pulp stem cells in hypoxic conditions
Source: Stem Cell Res Ther. 2019 Mar 12;10:89. doi: 10.1186/s13287-019-1192-2 (PMC6417198; doi:10.1186/s13287-019-1192-2)
Supplement: Supplementary file 3 — Table S3. The differentially expressed lncRNAs of hDPSCs in hypoxic and normoxic conditions. (DOCX 20 kb) [file 13287_2019_1192_MOESM3_ESM.docx]

**Table S3.** The differentially expressed lncRNAs of hDPSCs in hypoxic and normoxic conditions

| **Gene_Symbol** | **lncRNA Accession** | **Fold Change  ( hypoxia/ normoxia)** | **Regulation** | ***p*-value** |
| --- | --- | --- | --- | --- |
| RP11-540H22.2 | OTTHUMT00000053531 | 2.56 | up | 0.015 |
| LINC00707 | NR_038291 | 2.46 | up | 0.033 |
| linc-WDR7-4 | TCONS_00026337-XLOC_012684 | 1.96 | up | 0.025 |
| AF075003 | AF075003 | 1.80 | up | 0.021 |
| uc004elv.1 | BC036602 | 1.66 | up | 0.044 |
| MEG3 | uc004elv.1 | 1.66 | up | 0.015 |
| OTTHUMT00000086351 | OTTHUMT00000086351 | 1.65 | up | 0.021 |
| RP11-111D3.2 | OTTHUMT00000041582 | 1.62 | up | 0.008 |
| RP1-127L4.7 | OTTHUMT00000156190 | 1.61 | up | 0.013 |
| LINC00919 | NR_038233 | 1.60 | up | 0.017 |
| AF085958 | AF085958 | 1.59 | up | 0.011 |
| AC105393.1 | AX750575 | 1.56 | up | 0.037 |
| AX750575 | OTTHUMT00000322402 | 1.56 | up | 0.017 |
| linc-RND3-1 | TCONS_00003420-XLOC_002355 | 1.55 | up | 0.040 |
| linc-GRHPR | TCONS_00015974-XLOC_007346 | 1.54 | up | 0.021 |
| uc004aan.1 | uc004aan.1 | 1.52 | up | 0.047 |
| AF131798 | AF131798 | 1.51 | up | 0.010 |
| RP11-13E5.2 | OTTHUMT00000058182 | 1.51 | up | 0.035 |
| RP11-533E19.2 | AF087982 | 1.50 | up | 0.032 |
| AF087982 | OTTHUMT00000085293 | 1.50 | up | 0.007 |
| linc-TGFBR2-2 | TCONS_l2_00030513-XLOC_l2_015744 | -1.51 | down | 0.041 |
| linc-LAS1L | TCONS_00005976-XLOC_002606 | -1.51 | down | 0.023 |
| linc-TMEM72-5 | TCONS_00017820-XLOC_008470 | -1.52 | down | 0.025 |
| linc-ODZ4-1 | TCONS_00019719-XLOC_009516 | -1.53 | down | 0.003 |
| RP11-720D4.3 | ENST00000532606 | -1.53 | down | 0.040 |
| AC009404.2 | OTTHUMT00000129613 | -1.53 | down | 0.041 |
| linc-NHLH2-6 | TCONS_00001636-XLOC_000973 | -1.54 | down | 0.027 |
| linc-RGPD1 | TCONS_00004326-XLOC_002196 | -1.56 | down | 0.008 |
| linc-PBX1 | TCONS_00026472-XLOC_012800 | -1.57 | down | 0.032 |
| linc-YIPF5-4 | TCONS_00010520-XLOC_005040 | -1.57 | down | 0.023 |
| linc-CDH2-1 | ENST00000507857 | -1.57 | down | 0.044 |
| RP11-257A22.1 | TCONS_00001181-XLOC_000447 | -1.57 | down | 0.004 |
| RP11-90B9.2 | ENST00000560450 | -1.58 | down | 0.041 |
| uc003vcp.1 | uc003vcp.1 | -1.60 | down | 0.030 |
| uc002czn.2 | uc002czn.2 | -1.62 | down | 0.013 |
| linc-BPY2B-8 | TCONS_l2_00030955-XLOC_l2_015966 | -1.62 | down | 0.031 |
| PIR-FIGF | NR_037859 | -1.62 | down | 0.044 |
| linc-RPRM-7 | TCONS_00005257-XLOC_002366 | -1.66 | down | 0.034 |
| linc-STAC | AF339786 | -1.69 | down | 0.042 |
| LOC143188 | NR_015409 | -1.69 | down | 0.024 |
| AF339786 | TCONS_00005987-XLOC_002614 | -1.69 | down | 0.010 |
| RP3-413H6.2 | OTTHUMT00000039866 | -1.70 | down | 0.001 |
| BC018684 | BC018684 | -1.80 | down | 0.023 |
| RP11-402L1.11 | OTTHUMT00000047721 | -1.86 | down | 0.004 |
| STL | ENST00000439075 | -1.92 | down | 0.043 |
| linc-PPIL4 | TCONS_00011593-XLOC_005875 | -1.98 | down | 0.024 |
| uc003ubq.3 | uc003ubq.3 | -2.41 | down | 0.002 |
