# Supplementary material for: Analysis of the characteristics and expression profiles of coding and noncoding RNAs of human dental pulp stem cells in hypoxic conditions
Source: Stem Cell Res Ther. 2019 Mar 12;10:89. doi: 10.1186/s13287-019-1192-2 (PMC6417198; doi:10.1186/s13287-019-1192-2)
Supplement: Supplementary file 4 — Table S4. The differentially expressed miRNAs of hDPSCs in hypoxic and normoxic conditions. (DOCX 14 kb) [file 13287_2019_1192_MOESM4_ESM.docx]

| **Table S4.** The differentially expressed miRNAs of hDPSCs in hypoxic and normoxic conditions | | | | |
| --- | --- | --- | --- | --- |
| **miRNA Symbol** | **miRNA Accession** | **Fold change  ( hypoxia/ normoxia)** | **Regulation** | ***p*-value** |
| hsa-miR-210-3p | MIMAT0000267 | 2.13 | up | 0.032 |
| hsa-miR-140-3p | MIMAT0004597 | 1.78 | up | 0.042 |
| hsa-miR-3149 | MIMAT0015022 | 1.73 | up | 0.021 |
| hsa-miR-550a-5p | MIMAT0004800 | 1.56 | up | 0.016 |
| hsa-miR-192-5p | MIMAT0000222 | 1.56 | up | 0.022 |
| hsa-miR-34b-3p | MIMAT0004676 | 1.55 | up | 0.032 |
| hsa-miR-3916 | MIMAT0018190 | 1.53 | up | 0.025 |
| hsa-miR-379-3p | MIMAT0004690 | -1.51 | down | 0.016 |
| hsa-miR-410-3p | MIMAT0002171 | -1.66 | down | 0.021 |
| hsa-miR-6872-5p | MIMAT0027644 | -1.67 | down | 0.038 |
| hsa-miR-4685-3p | MIMAT0019772 | -1.75 | down | 0.003 |
| hsa-miR-6805-3p | MIMAT0027511 | -1.83 | down | 0.020 |
| hsa-miR-6744-5p | MIMAT0027389 | -2.04 | down | 0.016 |
| hsa-miR-7846-3p | MIMAT0030421 | -2.09 | down | 0.047 |
